# Diverse regulatory manners of human telomerase reverse transcriptase

**DOI:** 10.1186/s12964-019-0372-0

**Published:** 2019-06-11

**Authors:** Meng-Meng Jie, Xing Chang, Shuo Zeng, Cheng Liu, Guo-Bin Liao, Ya-Ran Wu, Chun-Hua Liu, Chang-Jiang Hu, Shi-Ming Yang, Xin-Zhe Li

**Affiliations:** 1 Department of Gastroenterology, Xinqiao Hospital, Third Military Medical University (Army Medical University), Chongqing, 400037 China; 2 Teaching evaluation center of Third Military Medical University (Army Medical University), Chongqing, 400038 China

**Keywords:** hTERT, Regulation, Transcription, Post-transcription, Post-translational modification

## Abstract

Human telomerase reverse transcriptase (hTERT) is the core subunit of human telomerase and plays important roles in human cancers. Aberrant expression of hTERT is closely associated with tumorigenesis, cancer cell stemness maintaining, cell proliferation, apoptosis inhibition, senescence evasion and metastasis. The molecular basis of hTERT regulation is highly complicated and consists of various layers. A deep and full-scale comprehension of the regulatory mechanisms of hTERT is pivotal in understanding the pathogenesis and searching for therapeutic approaches. In this review, we summarize the recent advances regarding the diverse regulatory mechanisms of hTERT, including the transcriptional (promoter mutation, promoter region methylation and histone acetylation), post-transcriptional (mRNA alternative splicing and non-coding RNAs) and post-translational levels (phosphorylation and ubiquitination), which may provide novel perspectives for further translational diagnosis or therapeutic strategies targeting hTERT.

## Background

Telomeres are located at the ends of chromosomes and their major functions are to maintain chromosomal integrity and genome stability [1]. Telomerase is a complex ribonucleoprotein and an essential reverse transcriptase which promotes the capping of eukaryotic telomere ends [2, 3]. The activity of telomerase is absent in most somatic tissues whereas it is commonly present in germ cells and some stem cells [4–6]. Dysregulation of telomerase results in its activation in approximately 90% of human cancers [7]. Importantly, cancer cells have acquired the ability to overcome replicative senescence and enhance growth ability via maintaining telomere length and activity. The differences between normal and cancer cells make telomerase an attractive cancer biomarker in clinical practice [8, 9]. The core human telomerase comprises of two essential components: the template RNA subunit, human telomerase RNA (hTERC), and the catalytic protein subunit, human telomerase reverse transcriptase (hTERT) [10, 11]. The expression and function of hTERT are crucial determinants of telomerase activity, which are strictly controlled by various molecule events in multiple layers. In this review, we summarize the diverse regulatory mechanisms of hTERT.

## Regulation of hTERT at transcriptional levels

Numerous researches have been performed to investigate the regulation of hTERT transcription through direct binding of transcription factors to the wild-type (WT) hTERT promoter. Canonical positive regulators include the multifunctional transcription factors c-MYC, NF-κB, and STAT3. c-MYC dimerizes with its partner MAX and binds to the E-box sequences on the hTERT core promoter region, which activates the transcription of hTERT [12–14]. Besides, c-MYC could cooperate with specificity protein 1 (SP1) to elevate hTERT transcription through their respective binding sites on the hTERT promoter region [15, 16]. In addition, a variety of factors regulate hTERT transcription by modulating c-MYC. For instance, p300 activates hTERT gene expression by interacting with and stabilizing c-MYC [17]. Sirtuin 1 (SIRT1) increases hTERT expression via upregulating FOXO3a-mediated activation of c-MYC expression in human umbilical cord fibroblast cells [18]. Aurora-A kinase promotes c-MYC expression thereby increasing hTERT expression [19]. As for NF-κB, it regulates hTERT gene transcription by direct binding to the proximal promoter region of hTERT [20–22] or by indirectly modulating the upstream regulators of hTERT [23, 24]. STAT3 also shows an important regulatory role in hTERT expression in various cancer cell lines [25, 26]. In addition to the positive regulators, the negative regulators of hTERT include E2F1 [27, 28] and MAD1 [29–31].

Interestingly, several transcription factors play dual roles in hTERT transcription. One representative is SP1. It has been reported that SP1 can activate hTERT expression by binding to five GC-box motifs at the hTERT promoter region in telomerase-positive cells [15, 32, 33]. Besides, SP1 could cooperate with c-MYC to bind to the promoter region of hTERT thereby upregulating hTERT [15, 16]. On the contrary, in telomerase-negative somatic cells, suppression of hTERT is due to the binding of SP1 to the proximal promoter region and recruitment of histone deacetylase (HDAC) proteins to the binding motifs [34, 35]. Histone deacetylation results in the silencing of hTERT transcription. Other factors that play duel effects on hTERT transcription include activator protein 1 (AP-1) [36, 37] and hypoxia-inducible factor 2-alpha (HIF2α) [38].

In addition to the common transcriptional regulation manners described above, genetic mutations and/or epigenetic modifications (methylation and acetylation) provide other regulatory modes to precisely alter the transcription of hTERT.

### Promoter mutations

#### Regulation and function

hTERT promoter mutations are frequently recurring events in many cancer types [39], including hepatocellular carcinoma (HCC), glioblastoma multiforme (GBM), thyroid carcinoma, urothelial cancer and melanoma [40–47]. However, they are rarely detected in lung, prostate, breast, gastrointestinal and kidney cancers and hematological malignancies [41, 47–49]. To date, two point mutations seem to be the most important. The C > T mutation takes place at the nucleotide residue − 146 or − 124 of the proximal promoter region upstream from the ATG start site in a mutually exclusive manner, which is termed − 146 C > T or − 124 C > T, respectively. These two somatic mutations give rise to the de novo generation of the E-twenty-six (ETS) family transcription factors consensus binding motifs [44, 50, 51]. The siRNA screening and subsequent verification experiments reveal that among the 13 ETS transcription factors, GABPA is the most positively related to the hTERT expression in hTERT promoter mutant (hTERT^Mut^) GBM cell lines [52]. Besides, bioinformatics prediction and subsequent experimental validation suggest that GABPA is specifically recruited to the mutation site of the hTERT promoter region during telomerase reactivation. GABPA knocking down dramatically decreases the activity of the mutant hTERT promoter without affecting that of the WT promoter, indicating that GABPA is the hTERT-regulating ETS factor that acts in a mutation-specific manner [52]. In addition, in multiple cancer cell lines that carry heterozygous mutant hTERT promoters, the mutant promoters display the active chromatin marker H3K4me2/3 and recruit the ETS family transcription factor GABPA/B1. On the contrary, the WT hTERT allele retains the epigenetic suppressive marker H3K27me3. These results suggest that only the mutant promoters are transcriptionally active and that one single base pair mutation can result in a dramatic epigenetic switch [53]. Interestingly, the selective upregulation of GABPB1 (but not of GABPA or GABPB2) in hTERT^Mut^ HCC cells could specifically enhance the levels of GABPA-bound genes (such as hTERT) other than other ETS transcription factor-regulated genes [52, 53]. Notably, some reports suggested that the − 146 C > T and − 124 C > T mutant hTERT promoters function differently and that they take effect depending on the context [54, 55]. In the background of the − 146 C > T mutation, a novel ETS binding motif promotes the dimerization and cooperative binding of ETS1 and p52 to the hTERT promoter region. However, this phenomenon is present in neither WT nor − 124 C > T hTERT promoters. Therefore, stabilization of the ETS-p52 complex on the − 146 C > T mutant hTERT promoter enhances hTERT expression in the context of non-canonical NF-κB signaling [55]. In addition to the canonical − 146 C > T and − 124 C > T mutations, two CC > TT tandem mutations (− 124/− 125 bp and − 138/− 139 bp) also produce the ETS transcription factor binding motifs, which probably heighten the transcription of hTERT [56, 57]. In conclusion, the ETS family transcription factors cooperate with the mutant hTERT promoters to enhance hTERT expression.

The hTERT promoter mutations also frequently accompany with the mutations of other oncogenic genes or tumor suppressors. For example, the co-occurrence of mutant hTERT promoter and TP53/RB1 mutations are found in bladder cancer (BC), suggesting a cooperative contribution to the BC progression [58]. Furthermore, hTERT promoter mutations are also closely associated with FGFR3 mutations in BC [59], or with BRAF/NRAS mutations in melanoma or papillary thyroid carcinoma (PTC) [60, 61].

#### Clinical significances

hTERT promoter mutations are tightly correlated with enhanced hTERT transcription and increased telomerase activity in tumors, which indicates that hTERT promoter mutations may be an essential mechanism of telomerase reactivation in cancers [45, 62–64]. In addition, hTERT promoter mutations are present in malignant tumors whereas they are absent in normal tissues, and hTERT promoter mutations are associated with poor survival or early diagnosis in various cancer types. The most representative cancer types are urothelial cancers because they harbor high frequencies of hTERT promoter mutations and can predict distant metastasis [46, 65–67]. Wang and colleagues detect the levels of the mutant hTERT promoter in urinary DNA derived from BC and renal pelvic cancer (RPC) patients before and after surgery, respectively. Their results show that the mutant hTERT promoter can serve as a urinary biomarker for diagnosis and recurrence surveillance [67]. Among BC patients, the hTERT promoter mutations are the most clinically related somatic lesions. Patients with hTERT promoter mutations showed poor prognosis in the absence of the allele of the rs2853669 polymorphism. Moreover, the mutations without this variant allele were highly correlated with the recurrence in patients with Tis, Ta, and T1 tumors [45].

**Fig. 1:**
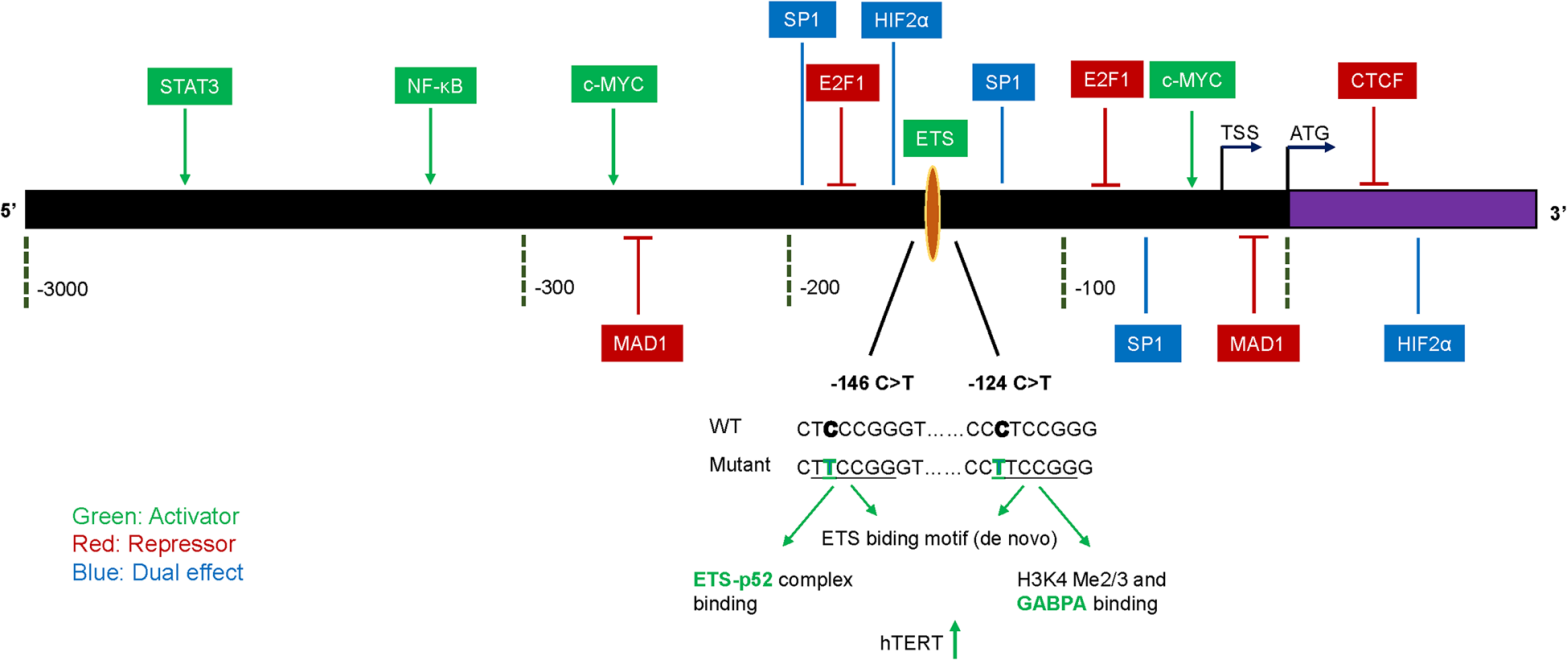
Transcription factors and promoter mutations that regulate the expression of hTERT. Green: activator; Red: repressor; Blue: dual effect

When examining thyroid nodule specimens prior to operation, it is found that all 9 specimens from thyroid cancer patients contain mutant hTERT whereas no hTERT promoter mutations are detected in 179 benign specimens, which reveals a 100% diagnostic specificity [68]. In addition, PTCs that concurrently harbor BRAF^V600E^ and hTERT promoter mutations (− 124 C > T) are more aggressive compared to either mutation alone, which defined a new genetic background with the worst clinicopathologic prognosis. Therefore, combinations using BRAF and hTERT promoter mutations may redirect the risk classifications of PTC [69, 70]. Among glioma patients, the relationship between hTERT promoter mutations and prognosis is relatively complicated, and additional factors such as tumor grade, and other genetic mutations can also make a difference. For example, the presence of − 146 C > T and − 124 C > T mutations is bound up with a shorter overall survival (OS) in high-grade glioma patients. However, OS was longer for low-grade glioma patients with these mutations [71]. In addition, patients with grade II and III gliomas who harbor only hTERT promoter mutations have a relatively poorer OS, in contrast, patients harboring both TERT and IDH mutations show good outcomes [72–74]. Nault et al. found that hTERT promoter mutation is the earliest as well as the most frequent genetic alteration in early HCC. Therefore, hTERT promoter mutation may be a novel biomarker predicting transformation of premalignant lesions into HCC [75]. Among melanoma patients, the co-existence of hTERT promoter and BRAF/NRAS mutations were related to 2-fold reduced disease-free and 5-fold reduced melanoma-specific survival [76]. Even in clear cell renal cell cancer (ccRCC) that harbored a relatively low frequency of hTERT promoter mutations compared to other cancers, Hosen et al. shows that ccRCC patients with hTERT promoter mutations in the absence of the rs2853669 polymorphism had the worst disease-specific survival [77]. Taken together, the mutant hTERT promoters may facilitate the malignant phenotypes of cancers and predict poor prognosis by upregulating the expression of hTERT. Transcription factors and promoter mutations that regulate the expression of hTERT are shown in Fig. 1.

### Promoter region methylation

As mentioned above, tumor types such as breast cancer and prostate cancer do not display high frequency of hTERT promoter mutations. Thus, there may be other regulatory mechanisms of hTERT transcription. Therefore, epigenetic mechanisms such as promoter region methylation or histone acetylation are likely to play effects. Traditionally, methylation of gene promoter region is often associated with gene expression silencing. Methylation of the first E-box CpG site (− 165 to − 160 bp) of hTERT promoter region and/or histone H3K9 hinders the binding of c-MYC and consequently decreases hTERT expression in HCC. When histone H3K9 is acetylated and the promoter region of hTERT is not methylated, c-MYC could enhance hTERT expression [78]. Besides, the hTERT promoter region is unmethylated in undifferentiated embryonic human teratocarcinoma (HT) cells. Retinoic acid (RA) treatment increases hTERT promoter region methylation and suppresses its expression. In addition, treatment with Trichostatin A (TSA) reactivates hTERT in only very early differentiating HT cells. In late differentiating cells, treatment with 5-AZA, but not TSA, upregulates the hTERT transcription, suggesting that histone deacetylation correlates with early hTERT expression inhibition and that hTERT promoter region methylation may maintain this silencing effect [79].

On the contrary, various studies have demonstrated that methylation within the regulatory element of the hTERT promoter region can enhance rather than weaken the expression of hTERT [80, 81], whereas demethylation decreases hTERT expression [82–84]. An example is the regulation of the hTERT promoter region by the repressive factor CTCF. Renaud et al. reveals that methylation of the hTERT promoter region plays a dual role inside and outside the CTCF-binding regions [83]. On the one hand, CTCF binds to the unmethylated first exon (+ 4 to + 39 bp) of hTERT thus repressing its transcription. Nevertheless, CTCF does not bind to the methylated first exon of hTERT. Besides, upon treatment with the demethylating agent 5-AZA, CTCF could interact with the hTERT promoter region and downregulate its mRNA level. These results suggest that hTERT promoter region methylation is essential for its transcription [83, 85, 86]. On the other hand, total methylation of the hTERT promoter region leads to full transcriptional repression, and the methylation cassette assay reveals that selective demethylation of a specific 110 bp sequence (− 183 to − 74 bp) within the core promoter region of hTERT significantly increases its transcription [83]. Thus, hypermethylation of most CpG islands restricts the binding of repressive factors and hypomethylation of specific regions allows the binding of activating factors. This dynamic equilibrium elaborately regulates hTERT promoter activation, which finally affects hTERT transcription. Another hTERT repressor, Wilms tumor protein (WT1), shows a methylation-sensitive binding manner to DNA sequences [87]. When its binding sequence is methylated, the binding affinity of WT1 is reduced. Therefore, methylation of the hTERT promoter region within the WT1 binding site (− 358 to − 349 bp) derepresses hTERT transcription [79]. Intriguingly, DNA methylation analysis shows that the hTERT promoter region around the transcriptional starting site (TSS) (− 200/− 150 to + 150 bp) is hypomethylated and associated with active chromatin marks, while the promoter region upstream of the TSS (− 650 to − 200/− 150 bp) is significantly hypermethylated [88, 89].

Several bioactive dietary, such as EGCG [90], Sulforaphane (SFN) [91] or Genistein [92], components display antitumor effects through regulating the methylation manner of the hTERT promoter region. EGCG weakens methylation of the hTERT promoter region in a time-dependent manner in MCF-7 breast cancer cells. Besides, EGCG induces demethylation at the putative E2F1 binding sites within the hTERT promoter region and increases the binding capacity of E2F1 at these sites, which may be a proposed mechanism for downregulation of hTERT by EGCG [90]. SFN treatment downregulates hTERT and reduces DNA methyltransferase 1 (DNMT1) and DNA methyltransferase 3a (DNMT3a) in human breast cancer cells. DNMTs reduction leads to site-specific CpG demethylation primarily in the first exon of the hTERT gene, which facilitates the association of CTCF with hTERT [91]. In addition, Genistein or EGCG inhibits transcription of hTERT via suppressing DNMTs-mediated hTERT promoter region methylation in breast cancer cells [90, 92, 93].

### Histone acetylation

Histone acetyltransferases (HATs) and HDACs induce the acetylation and deacetylation of chromatin histone or non-histone proteins respectively, thereby modulating the expression or function of their targets. Histone acetylation or deacetylation has been shown to be associated with hTERT transcription and they seem to be a general underlying characteristic of human normal cells, telomerase-negative immortal cell lines and cancer cells [34, 94, 95]. Besides, core histones hyperacetylation at the hTERT promoter region is also related to its transcription in multiple human cells [14, 96]. Treating telomerase-negative cells with the histone deacetylase inhibitor (HDACi) TSA causes hTERT mRNA upregulation and telomerase activation. In addition, MAD-mediated repression of the hTERT promoter requires histone deacetylase activity, whereas TSA-mediated derepression is not dependent on E-boxes [94]. Moreover, Won and colleagues show that SP1 and SP3 can interact with the hTERT promoter region, and recruit HDAC to deacetylate the nucleosomal histones and silence hTERT transcription in normal human somatic cells [35].

Several agents can regulate the hTERT promoter activity via acetylation or deacetylation. The HDACi TSA enhances histone acetylation at the proximal promoter region of hTERT, and directly transactivates the hTERT gene and upregulates hTERT expression. In addition, the effect of TSA is dependent on SP1 motifs [34]. Interestingly, Kretzner et al. reports that hTERT expression is reduced by the combination of aurora kinase inhibitors with the HDACi vorinostat in lymphoma cells. The reasons for the above discrepancy may imply the cell-type specificity and cellular context [97]. ChIP assay of the hTERT promoter region shows that SFN upregulates the level of active chromatin markers (such as acetyl-H3K9 and acetyl-H4), whereas it downregulates the level of suppressive chromatin markers (such as trimethyl-H3K9 and trimethyl-H3K27). The hyperacetylation induced by SFN accelerates the interaction of hTERT repressors (such as MAD1 and CTCF) with hTERT regulatory region. Silencing of CTCF rescues the SFN-induced hTERT transcription inhibition in breast cancer cells [91]. In another study, Meeran et al. reveals that both EGCG, the major component of green tea polyphenols, and its prodrug pro-EGCG decreases hTERT transcription in breast cancer cells. The reason is partially due to the hypomethylation of the hTERT promoter region and histone deacetylation [93]. EGCG and pro-EGCG decrease the levels of acetyl-H3, acetyl-H3K9, and acetyl-H4 at the hTERT promoter region. Structural alterations of chromatin facilitate the binding of several hTERT repressors such as E2F1 and MAD1 to the promoter region of hTERT. Silencing of E2F1 and MAD1 reverse the pro-EGCG-mediated hTERT downregulation [90, 93].

Notably, methylation and acetylation are often crossed in the regulation of hTERT expression. Distinct modifications within the chromatin of the hTERT promoter correlate with different hTERT expression levels. For example, low hTERT expression is related to hypoacetylation of histone H3 and H4 and methylation of histone H3K9 in ALT cell lines. In contrast, hyperacetylation of H3 and H4, and methylation of H3K4 are associated with high hTERT expression in telomerase-positive tumor cells. However, methylation of H4K20 is specific to hTERT promoter region but is not involved in gene expression in ALT cells [98]. Besides, histone methyltransferase, SET and MYND domain-containing protein 3 (SMYD3), directly transactivates the hTERT gene. The association of SMYD3 with the hTERT promoter region is required for maintaining histone H3K4 trimethylation, which results in constitutive and inducible hTERT expression. SMYD3 downregulation abrogates trimethylation of H3K4, diminishes acetylation of histone H3, and alleviates the occupancy of c-MYC and SP1 at the hTERT promoter region, ultimately resulting in reduced hTERT transcription [99]. In addition, depletion of SIRT1, a versatile HDAC, causes little change in CpG methylation patterns of the hTERT promoter region, and also does not affect hTERT mRNA stability. However, SIRT1 silencing is involved in a remarkable increase in acetylated histone H3K9 and a decrease in trimethyl histone H3K9 at the hTERT promoter region. These results indicate that SIRT1 regulates not only histone acetylation but also methylation of the hTERT promoter region [100]. Intriguingly, in addition to the direct epigenetic modification of hTERT, SIRT1 could also deacetylate c-MYC and promote the interaction of c-MYC with its coactivator MAX, which facilitates the transactivation activity on the hTERT promoter region [101]. The HDACi TSA could induce site-specific CpGs demethylation on the hTERT promoter region by downregulating DNMT1. ChIP assays show that TSA-mediated 31st-33rd CpGs demethylation promotes the binding of CTCF to the hTERT promoter region, which results in the hTERT transcription repression in colon cancer cells [102].

## Regulation of hTERT at post-transcriptional levels

### Alternative mRNA splicing

There are a plenty of alternative splicing variants for hTERT transcripts, but the regulatory mechanisms and functions are not totally clear. Among them, the function of two hTERT splicing variants, the α-deletion and β-deletion forms have been well studied [103–105]. The α-deletion form causes the loss of reverse transcriptase (RT) motif A and displays dominant negative effects on telomerase activity in human lung fibroblast cells and prostate cancer cells. However, the other two splicing variants (α + β-deletion and β-deletion forms) do not display any phenotype using the same models [103]. The β-deletion variant is the most extensively expressed hTERT splicing variant in stem cells and cancer cells, and it is degraded by nonsense-mediated decay [105, 106]. The β-deletion form creates a frameshift by skipping the exons 8 and 9, and encounters a premature stop codon in exon 10. Splicing of the β-deletion variant is mediated by the splice factors HNRNPL, HNRNPH2, and SRSF11. The β-deletion hTERT overexpression competes with the WT hTERT to bind to hTERC, thereby suppressing telomerase activity. In addition, overexpressing β-deletion protein protects breast cancer cells from the cisplatin-induced apoptosis. These results reveal that the β-deletion hTERT splicing variant possesses a novel oncogenic function independent of maintaining telomere activity [105].

Different to most alternative splicing regulatory mechanisms, the splicing of hTERT mRNA is regulated by long-range interactions rather than by the intronic/exonic elements close to the splice sites [107]. A block of 26 short repeats with a 38 nt consensus sequence downstream of exon 6 (approximately 1900 nt) within the hTERT gene is called a block of repeats in intron 6 (B6, block 6), and a direct repeat (256 nt) in intron 6 or a direct repeat (256 nt) in intron 8 is called DR6 or DR8, respectively [107]. The number of B6 repeats varies from 18 to 38 repeats among different individuals, and altered repeats can cause diseases including cancers. This potential association between B6 repeats and cancer development requires further investigations [108]. B6 is required for β-deletion splicing, but DR6 and DR8 are not sufficient to create the skipping of exons 7 and 8. However, they regulate the role of B6 to shift splicing to nonfunctional variants. Exchanging the positions of DR6 and DR8 or replacing each other reciprocally does not change the splicing patterns of hTERT. These results suggest that the intronic location instead of the sequence differences between DR6 and DR8 could determine their effect on alternative splicing, and that manipulating hTERT splicing may provide an avenue for chemotherapy and regenerative medicine [107].

Many factors can influence hTERT splicing. TGF-β1 could downregulate c-MYC and subsequently decrease hTERT expression in human skin keratinocytes. Intriguingly, TGF-β1 still inhibits the telomerase activity without hindering hTERT transcription by decreasing the full-length (FL) hTERT transcript and retaining high levels of the inactive β-deletion variant. Upon TGF-β1 removal, the changes in the hTERT mRNA splicing pattern disappear, suggesting that alternative splicing may be a novel mechanism for TGF-β1-mediated regulation of telomerase [109]. Besides, knockdown of neuro-oncological ventral antigen 1 (NOVA1) leads to a switch in hTERT splicing from the FL form towards a non-catalytic alternative variant and reduces telomerase activity by approximately 50%. NOVA1 downregulation also significantly decreases cancer cell growth both in vitro and in vivo. What’s more, NOVA1 facilitates the inclusion of exons in the hTERT reverse transcriptase domain, which produces FL hTERT transcripts. The β-deletion hTERT variant increases when NOVA1 decreases [110]. Interestingly, targeting NOVA1 can also downregulate the hTERT transcription, a possible reason is that NOVA1 may regulate the upstream transcription factors of hTERT in cancer cells [111]. Mechanistic research shows that when NOVA1 binds to the DR8 region of hTERT mRNA, it functions as a splicing enhancer to increase the FL hTERT mRNAs by including the exons 7 and 8. These data verify the role of NOVA1 and DR8 in balancing the production of FL or spliced hTERT variants [110].

The alternative splicing of hTERT mRNA may provide a regulatory mechanism that could not be accomplished at the transcriptional level, and manipulating hTERT splicing may provide new insights into cancer therapeutics [107].Such subtle modulation may be especially important in hematopoietic stem cells where maintaining low telomerase activity might slow down but not abolish the telomere shortening [112]. Besides, alternative splicing can decease telomerase activity while exempting hTERT downregulation under conditions such as carcinogenesis [113, 114] or differentiation [115]. Regulation of hTERT splicing patterns is involved in several biological events, such as hypoxia or determination of immune cell fate. For instance, it has been known that HIF1α elevates hTERT expression by activating its promoter [116, 117]. Hypoxia enhances the transcriptional activity of both hTERT and hTERC gene promoters. Interestingly, regulation of the hTERC gene is at the transcriptional level, while regulation of the hTERT gene is by an alternative splicing mechanism, which is related to a splicing pattern switch to the active WT hTERT form rather than simply upregulating total hTERT expression [118]. Besides, hypoxia causes high levels of nuclear hTERT and cell survival, and maintains the undifferentiated state. The splicing pattern of hTERT is distinct under different oxygen concentrations in human embryonic stem cells (hESCs). Hypoxia microenvironment decreases the FL and α-deletion hTERT, while does not change the levels of β-deletion or the α + β-deletion variants. In addition, steric blocking of α-deletion and β-deletion splicing variants changes the hTERT variant patterns and results in hESCs differentiation [119]. Moreover, overexpressing the apoptotic endonuclease EndoG in human CD4^+^ T cells reduces the expression of the active FL hTERT variant and enhances the expression of the inactive β-deletion variant, which results in the decreased telomerase activity and a replicative senescent state [120].

### Non-coding RNAs

#### microRNA

It has been reported that hTERT can be regulated by different microRNAs (miRNAs) in many types of cancers (Table 1). MiRNAs influence the expression of hTERT proteins mainly by directly binding to its 3′-untranslated region (3′-UTR). This changes the tumor cell proliferation, apoptosis, migration, invasion and chemosensitivity. For instance, miR-138 is significantly downregulated in anaplastic thyroid carcinoma (ATC) cell lines. Overexpression of miR-138 reduces the hTERT protein level through binding to its 3′-UTR [121]. Besides, miR-615-3p locates in an intron of the HOXC5 gene and negatively regulates hTERT by targeting its 3′-UTR. Furthermore, miR-615-3p and HOXC5 make a feed-forward loop that orchestrates transcriptional and post-transcriptional suppression of hTERT during cellular differentiation [134]. Among the known miRNAs that regulate hTERT, miR-1182 has dual effects in downregulating hTERT. On the one hand, it targets the open reading frame (ORF) of hTERT, thereby reducing proliferation and metastasis of gastric cancer cells. On the other hand, it directly targets the 3′-UTR of hTERT to enhance the chemosensitivity of bladder cancer cells [126, 127]. In addition to direct targeting, miRNAs can regulate hTERT indirectly via influencing the upstream regulators (transcription factors, etc.) of hTERT. For example, miR-34a targets FOXM1 and c-MYC, both of which can activate hTERT transcription and subsequently modulate telomerase activity and cellular senescence [133].

**Table 1:** The MiRNAs that regulate hTERT

MiRNA	Mechanism	Cancer cell types	Ref.
Negative regulators			
miR-138	targeting the 3′-UTR of hTERT	anaplastic thyroid carcinoma (ATC)	[121]
miR-138-5p and miR-422a	potentially inhibits hTERT	colorectal cancer (CRC)	[122]
miR-299-3p	targeting the 3′-UTR of hTERT	laryngeal cancer	[123]
miR-512-5p	targeting the 3′-UTR of hTERT	head and neck squamous cell carcinoma (HNSCC)	[124]
miR-661	targeting the 3′-UTR of hTERT	glioma cells	[125]
miR-1182	targeting the open reading frame (ORF) of hTERT	gastric cancer (GC)	[126]
miR-1182	targeting the 3′-UTR of hTERT	bladder cancer (BC)	[127]
miR-1207-5p and miR-1266	targeting the 3′-UTR of hTERT	gastric cancer (GC)	[128]
miR-491-5p	targeting the 3′-UTR of hTERT	cervical cancer	[129]
miR-133a, miR-138, and miR-491	targeting the 3′-UTR of hTERT	cervical cancer	[130]
miR-532 and miR-3064	targeting the 3′-UTR of hTERT	ovarian cancer	[131]
miR-498	targeting the 3′-UTR of hTERT	ovarian cancer	[132]
miR-34a	targeting the FOXM1/c-MYC pathway	hepatocellular carcinoma (HCC)	[133]
miR-615-3p	targeting the 3′-UTR of hTERT	multiple kinds of cancers	[134]
Positive regulators			
miR-346	competing with miR-138	cervical cancer	[135]
miR-19b	inhibition of PITX1	melanoma	[136]
miR-202	via the MXD1-MYC/MAX pathway	pancreatic cancer	[137]
miR-21	via the PTEN/ERK1/2 pathway	colorectal cancer (CRC)	[138, 139]

Typically, miRNAs downregulate hTERT by targeting its 3′-UTR. Unconventionally, there are also miRNAs that can positively regulate the expression of hTERT. For instance, in cervical cancer cells, miR-346 and miR-138 compete with each other to bind the common 3′-UTR region of hTERT mRNA. miR-138 suppresses the expression of hTERT in an AGO2-dependent manner. In contrast, miR-346 can upregulate hTERT [135]. The mechanism lies in GRSF1 binding to the miR-346 middle sequence motif which facilitates to recruitment of hTERT mRNA to ribosomes and promotes hTERT mRNA translation in an AGO2-independent manner. Interestingly, replacing the middle sequence of miR-138 with that of miR-346 upregulates hTERT in a GRSF1-dependent manner [135]. PITX1 is a suppressor gene of hTERT, and PITX1 is a direct target of miR-19b. Therefore, miR-19b enhances the expression of hTERT by alleviating the inhibitory effect of PITX1 on hTERT [136]. MXD1 is the target gene of miR-202 and pre-miR-202 overexpression decreases the level of MXD1. Besides, MXD1 binds to the promoter region of hTERT and reduces c-MYC binding to the hTERT promoter region, leading to the decreased hTERT mRNA expression. These findings suggest that miR-202 may target MXD1 to increase hTERT expression [137]. In addition, it has been reported that PTEN is a miR-21 target gene in CRC cell lines [138]. Further study reveals that miR-21 positively regulates hTERT expression through the PTEN/ERK1/2 signaling pathway. Moreover, hTERT inversely correlates with PTEN in high miR-21 RNA-expressing CRC tissues [139]. The miRNAs that negatively or positively regulate the expression of hTERT are listed in Table 1 [121–139].

#### Long non-coding RNA

Long non-coding RNAs (lncRNAs) are a set of transcripts longer than 200 nt with no or limited protein coding function. LncRNAs display their functions via different manners and regulation of gene expressions by lncRNAs is emerging as a novel and critical way [140, 141]. The hTERT antisense promoter-associated RNA (hTAP-AS) is an antisense transcript in the promoter region of hTERT which is located in both the cytoplasm and the nucleus. The expression of hTAP-AS is negatively related with hTERT in multiple human tumor types. Overexpression of hTAP-AS decreases hTERT while knockdown of hTAP-AS increases hTERT. Interestingly, the hTERT promoter mutation (− 124 C > T) remarkably upregulates hTERT but not hTAP-AS [142]. As the star molecules, both of the lncRNA RNA HOTAIR and MALAT1 can occupy the promoter region of estrogen target genes such as hTERT under basal conditions. Interestingly, estrogen markedly enhances the recruitment of HOTAIR while it inhibits the recruitment of MALAT1 onto hTERT promoter region. Besides, interfering HOTAIR delays the response of estrogen to hTERT while MALAT1 depletion increases hTERT mRNA levels in prostate cancer cells [143]. In esophageal carcinoma cells, treatment with β-elemene significantly suppresses cell proliferation and the level of hTERT while dramatically upregulates the lncRNA CDKN2B-AS1. When transfection with the siRNA against CDKN2B-AS1, the cell proliferation rate and hTERT expression are increased in the presence of β-elemene, indicating that the lncRNA CDKN2B-AS1-mediated downregulation of hTERT may play a crucial role in the anticancer effect of β-elemene [144]. As for the lncRNA BC032469, it is highly expressed in tissues of gastric cancer patient and predicts a poor prognosis. BC032469 serves as a competitive endogenous RNA (ceRNA) to suppress miR-1207-5p-mediated hTERT inhibition. Thus, silencing BC032469 remarkably inhibits proliferation of gastric cancer cells in vitro and in vivo [145]. In a study by Pu et al., the lncRNA RNA CUDR forms a complex with Cyclin D1 to enhance the expression of H19. Overexpression of H19 enhances the interaction between hTERT and TERC, and weakens the association between hTERT and TERRA, thereby heightening telomerase activity. Besides, hTERT upregulation plays an important role in CUDR-mediated liver cancer cell stemness [146]. Redon and colleagues find that repeats (5′-UUAGGG-3′) of the large non-coding RNA telomeric repeat containing RNA (TERRA) binds to hTERT and interacts with the telomerase RNA template hTERC through a base pairing mechanism. TERRA contacts the hTERT independently of hTERC. This study indicates that TERRA is a natural ligand and negative regulator of human telomerase [147].

In addition to traditional lncRNA RNAs, circular RNAs (circRNAs) represent a new direction of gene regulation. The circular RNA hsa_circ_0020397 upregulates the expression of hTERT by sequestering miR-138 (the negative regulator of hTERT). The pro-invasion effect of hsa_circ_0020397 is inhibited when hTERT is silenced, suggesting that the circRNA hsa_circ_0020397 promotes cancer cell invasion partially by regulating the expression of miR-138 target genes, such as hTERT [148]. The lncRNAs that regulate the expression (activity) of hTERT (telomerase) are listed in Table 2 [142–148].

**Table 2:** The lncRNAs that regulate hTERT

LncRNA	Effects on hTERT/telomerase	Mechanism	Human cell model	Ref.
hTAP-AS	hTERT expression ↓	not stated	embryonic kidney cells	[142]
HOTAIR	positive effect on hTERT in respond to estrogen	regulates the estrogen target genes positively by estrogen-modulated chromatin remodeling	prostate cancer cells	[143]
MALAT1	hTERT transcription ↓	functions on hTERT promoter	prostate cancer cells	[143]
CDKN2B-AS1	hTERT expression ↓	not stated	esophageal squamous carcinoma cells	[144]
BC032469	hTERT expression ↑	functions as a sponge for miR-1207-5p	gastric cancer cells	[145]
TERRA	functions as a telomerase ligand and direct telomerase inhibitor	TERRA base repeats pair with hTERC, TERRA contacts with hTERT protein	embryonic kidney cells	[147]
CUDR	the binding of hTERT/hTERC ↑ the binding of hTERT/TERRA ↓ telomerase activity ↑	the CUDR-CyclinD1 complex enhances the expression of H19	liver cancer stem cells	[146]
hsa_circ_0020397	hTERT expression ↑	inhibits miR-138 activity and its targets	colorectal cancer cells	[148]

↑ Promoting effect, ↓ Suppressing effect

## Regulation of hTERT protein at post-translational levels

Similar to other molecules, the post-translational modifications (PTMs) of hTERT could influence its protein stability, subcellular localization and subsequently change its protein levels or the telomerase activity. Currently, the post-translational regulations of hTERT mainly focus on phosphorylation and ubiquitination.

### Phosphorylation

Several studies have revealed that hTERT can be modified by phosphorylation. A prominent effect of phosphorylation is the cellular localization change of hTERT. The most reported kinases that phosphorylate hTERT are AKT, PKC and SRC kinases.

As a multifunctional kinase, AKT kinase phosphorylates and regulates hTERT in various ways. The hTERT peptide (^817^AVRIRGKSYV^826^) could be phosphorylated by the activated AKT kinase in vitro, which plays a crucial role in maintaining the telomere length and heightening telomerase activity [149]. Besides, AKT-mediated hTERT phosphorylation at serine 227 plays a significant role in directing its nuclear translocation. Interestingly, Ser^227^ phosphorylation of hTERT is equivalently necessary with the bipartite nuclear localization signal (NLS) motifs for efficient hTERT nuclear localization and immortalization of human foreskin fibroblast cells [150]. Furthermore, the Ser^227^ phosphorylation by AKT increases the binding affinity of hTERT with the nuclear import receptors importin-α, which promotes nuclear import of hTERT, thereby increasing telomerase activity [151]. In addition, hTERT phosphorylation by the PKC isoenzymes α, β, δ, ε and ζ is vital for maintaining telomerase holoprotein integrity in head and neck cancer cells, which finally results in telomerase activation and oncogenesis. Inhibition of telomerase by dephosphorylating PKC significantly chemosensitizes cisplatin [152]. Interestingly, in human breast cancer cells, although all PKC isoforms may potentially regulate telomerase activity, only PKCα interacts with partially purified telomerase. PKCα phosphorylates while PP2A dephosphorylates hTERT and human telomerase-associated protein 1 (hTEP1), and the hTERT/hTEP1 phosphorylation status by reversible regulation of PKCα and PP2A dramatically determines the telomerase activity in breast cancer cells [153]. These evidences imply that PKC differentially regulates hTERT in distinct models. Moreover, upon oxidative stress, hTERT translocates from nuclear to cytoplasm. This process depends on GTPase Ran through the export receptor CRM1 as well as tyrosine 707 (Tyr^707^) phosphorylation by the SRC kinase family. The specific SRC kinase inhibitor PP1 weakens hTERT nuclear export and decreases the antiapoptotic function of hTERT [154]. Meanwhile, the SRC kinase colocalizes with hTERT in mitochondria in primary human endothelial cells. During H_2_O_2_ stimulation, AKT1 is inactivated and the SRC kinase is activated. In the meantime, mitochondrial hTERT is decreased, which depends on SRC kinase-mediated Tyr^707^ phosphorylation [155]. As a protein tyrosine phosphatase that counteracts the function of the SRC kinase family, SHP-2 could complex with hTERT. Overexpression of wild-type, but not catalytically inactive, SHP-2 suppresses H_2_O_2_-induced hTERT nuclear export via regulating Tyr^707^ phosphorylation of hTERT [156]. The c-ABL tyrosine kinase, a DNA damage effector, could also induce tyrosine phosphorylation of hTERT upon ionizing radiation. c-ABL-mediated hTERT phosphorylation finally represses telomerase activity [157]. Interestingly, inhibition of c-ABL does not prevent H_2_O_2_-induced hTERT tyrosine phosphorylation [154]. Liu et al. reveals that during human CD4^+^ T lymphocytes activation, CD4^+^ T cells regulate telomerase function independent of the hTERT protein levels, nevertheless, hTERT phosphorylation and subsequent nuclear translocation may play an important role in regulating telomerase function [158]. In addition, high throughput screening suggests that there may be other potential phosphorylation sites within the hTERT protein, which warrants further investigations [159, 160].

### Ubiquitination

To date, there are only few reports that have investigated the ubiquitination modifications of hTERT. Kim et al. shows that breaking HSP90 function with geldanamycin induces ubiquitination and subsequent proteasome-mediated degradation of hTERT. As an E3 ubiquitin ligase, Makorin RING finger protein 1 (MKRN1) mediates the ubiquitination of hTERT. Overexpression of MKRN1 could promote hTERT degradation and downregulate telomerase activity and telomere length [161]. In addition, Oh et al. reveals that the E3 ligase HDM2 could polyubiquitinate hTERT mainly at the five lysines in its N-terminus, which further leads to its proteasome-dependent degradation. HDM2 depletion or transfection with the HDM2-resistant hTERT mutant reinforces the cellular antiapoptotic effects [162]. Moreover, CHIP and HSP70 associate with hTERT and repress its nuclear translocation by disaggregating hTERT with p23 rather than HSP90. CHIP overexpression promotes degradation of hTERT in the cytoplasm, thus suppressing the telomerase activity. Notably, CHIP can regulate the hTERT chaperone complexes in a cell cycle-dependent manner [163]. The currently reported PTMs of hTERT are listed in Table 3 [149–158, 161–163].

**Table 3:** The PTMs of hTERT

Enzyme	Site	Effects on hTERT/telomerase	Human cell model	Ref.
PTMs: Phosphorylation				
AKT	Ser 824	telomerase activity ↑	melanoma cells	[149]
AKT	Ser 227	hTERT nuclear translocation ↑ cell immortalization ↑	lung carcinoma cells and foreskin fibroblast cells	[150]
AKT	Ser 227	hTERT binding with importin-α ↑ hTERT nuclear import ↑ telomerase activity ↑	breast cancer cells	[151]
PKCα, β, δ, ε, ζ	Unclear	hTERT association with HSP90 ↑ telomerase holoprotein integrity ↑ telomerase activation ↑ chemosensitivity to cisplatin ↓	head and neck cancer cells	[152]
PKCα	Unclear	telomerase activity ↑	breast cancer cells	[153]
SRC	Tyr 707	cytoplasm translocation ↑ antiapoptotic function ↑	embryonic kidney cells	[154]
SRC	Tyr 707	mitochondrial hTERT ↓	umbilical and vein endothelial cells	[155]
c-ABL	Tyr	telomerase activity ↓	embryonic kidney cells and breast cancer cells	[157]
Unclear	Unclear	nuclear translocation ↑	CD4^+^ T cells	[158]
PTMs: Dephosphorylation				
SHP-2	Tyr 707	SRC kinase function↓ H2O2-induced hTERT nuclear export ↓nuclear telomerase activity ↑	endothelial cells	[156]
PTMs: Ubiquitination				
MKRN1	Unclear	hTERT degradation ↑ telomerase activity ↓ telomere length ↓	lung carcinoma cells	[161]
HDM2	N-Lys 78, 94, 236, 339, 348	hTERT degradation ↑ protection against apoptosis ↓	colon cancer, lung carcinoma, osteosarcoma and embryonic kidney cells	[162]
CHIP	Unclear	hTERT associating with p23 ↓ hTERT nuclear translocation ↓ hTERT degradation ↑	lung carcinoma cells	[163]

↑ Promoting effect, ↓ Suppressing effect

## Conclusions and future investigations

As the core subunit of telomerase, hTERT is normally expressed in highly self-renewal cells and absent in normal somatic cells. Deregulations of hTERT significantly promote cancer initiation and development. Up to now, a large range of mechanisms for hTERT regulation in both normal and cancer cells have been revealed, which have provided significant insights into the hTERT-based therapeutics. Our review has summarized different layers of hTERT regulation, including transcriptional (promoter mutation, promoter region methylation and histone acetylation), post-transcriptional (alternative splicing, non-coding RNAs) and post-translational levels (phosphorylation and ubiquitination), which may provide comprehensive perspectives for further basic and translational investigations regarding hTERT. Interestingly, these mechanisms are not isolated, but interact with each other. For instance, binding of the transcription factors to the hTERT promoter region could influence the alternative splicing of hTERT transcripts [116, 117, 119]. Besides, the hTERT promoter region methylation pattern can affect the binding of several transcription factors to the hTERT promoter region [79, 83]. Therefore, more studies are needed to fully clarify the possible crosstalk between these distinct mechanisms, and a better understanding of hTERT regulation elements are crucial for providing novel approaches in treating cancers.

A plenty of studies have illustrated the significant roles of hTERT regulation in human cancers. However, numerous questions still remain to be clarified. For example, what causes the significantly different frequencies of hTERT promoter mutation between distinct cancer types. In addition, although the mutant hTERT promoter sometimes coexists with other mutations, the detailed relationships (upstream or downstream) and the corresponding regulatory mechanisms are still unknown. Furthermore, whether mutant hTERT promoter targeted strategies are sufficient to suppress telomerase activity in cancer therapeutics also needs further investigations. At another layer, the post-translational modifications of hTERT dramatically influence the protein stabilization and localization of hTERT. Notably, there are few studies about the methylation, acetylation or SUMOylation modifications of the hTERT protein, and studies in these fields require further deep investigations.

Due to its critical roles in carcinogenesis and cancer progression, hTERT-based diagnosis and therapies have emerged and are worthy of expectations. The mutant hTERT promoter is a relatively unique feature in cancer mutations. Whether it can be a diagnostic biomarker used in liquid biopsies, such as detecting in exosomes or in circulating tumor cells, needs further explorations. In addition, hTERT-based agents [164, 165], peptide epitopes [166, 167] or suicide gene therapy [168] may have potential therapeutic effects. However, challenges still remain, because large-scale preclinical studies on drug efficacy, safety and tolerance of patients should be strictly examined. Another critical question that remains is that the intact crystal structure of hTERT is still not fully illuminated, which limits the precise structure-based drug designs for hTERT inhibitors. Taken together, further studies that elucidate these questions may accelerate the application of hTERT-based cancer diagnosis as well as therapeutics.

## Abbreviations

3′-UTR: 3′-untranslated region
AP-1: Activator protein 1
ATC: Anaplastic thyroid carcinoma
BC: Bladder cancer
ccRCC: Clear cell renal cell cancer
ceRNA: Competitive endogenous RNA
circRNAs: Circular RNAs
DNMT1: DNA methyltransferase 1
DNMT3a: DNA methyltransferase 3a
ETS: E-twenty-six
FL: Full-length
GBM: Glioblastoma multiforme
HATs: Histone acetyltransferases
HCC: Hepatocellular carcinoma
HDAC: Histone deacetylase
HDACi: Histone deacetylase inhibitor
hESCs: Human embryonic stem cells
HIF2α: Hypoxia-inducible factor 2-alpha
hTAP-AS: hTERT antisense promoter-associated RNA
hTEP1: Human telomerase-associated protein 1
hTERC: Human telomerase RNA
hTERT: Human telomerase reverse transcriptase
lncRNAs: Long non-coding RNAs
MKRN1: Makorin RING finger protein 1
NLS: Nuclear localization signal
NOVA1: Neuro-oncological ventral antigen 1
ORF: Open reading frame
OS: Overall survival
PTC: Papillary thyroid carcinoma
PTMs: Post-translational modifications
RA: Retinoic acid
RPC: Renal pelvic cancer
RT: Reverse transcriptase
SFN: Sulforaphane
SIRT1: Sirtuin 1
SMYD3: SET and MYND domain-containing protein 3
SP1: Specificity protein 1
TSA: Trichostatin A
TSS: Transcriptional starting site
WT: Wild-type
WT1: Wilms tumor protein

## Authors’ information

Jie M., Chang X., Yang S. and Li X. are all physician scientists.
